# Detection of abnormal resting-state networks in individual patients suffering from focal epilepsy: an initial step toward individual connectivity assessment

**DOI:** 10.3389/fnins.2014.00419

**Published:** 2014-12-23

**Authors:** Christian L. Dansereau, Pierre Bellec, Kangjoo Lee, Francesca Pittau, Jean Gotman, Christophe Grova

**Affiliations:** ^1^Multimodal Functional Imaging Lab, Biomedical Engineering Department, McGill UniversityMontreal, QC, Canada; ^2^Neurology and Neurosurgery Department, Montreal Neurological Institute, McGill UniversityMontreal, QC, Canada; ^3^Centre de Recherche de l'Institut Universitaire de Gériatrie de Montréal, Functional Neuroimaging Unit, Université de MontréalMontreal, QC, Canada; ^4^Department of Computer Science and Operations Research, University of MontrealMontreal, Quebec, Canada; ^5^Physics Department, PERFORM Center, Concordia UniversityMontreal, QC, Canada

**Keywords:** functional connectivity, resting state fMRI, focal epilepsy, single subject design, outlier detection

## Abstract

The spatial coherence of spontaneous slow fluctuations in the blood-oxygen-level dependent (BOLD) signal at rest is routinely used to characterize the underlying resting-state networks (RSNs). Studies have demonstrated that these patterns are organized in space and highly reproducible from subject to subject. Moreover, RSNs reorganizations have been suggested in pathological conditions. Comparisons of RSNs organization have been performed between groups of subjects but have rarely been applied at the individual level, a step required for clinical application. Defining the notion of modularity as the organization of brain activity in stable networks, we propose Detection of Abnormal Networks in Individuals (DANI) to identify modularity changes at the individual level. The stability of each RSN was estimated using a spatial clustering method: Bootstrap Analysis of Stable Clusters (BASC) (Bellec et al., 2010). Our contributions consisted in (i) providing functional maps of the most stable cores of each networks and (ii) in detecting “abnormal” individual changes in networks organization when compared to a population of healthy controls. DANI was first evaluated using realistic simulated data, showing that focussing on a conservative core size (50% most stable regions) improved the sensitivity to detect modularity changes. DANI was then applied to resting state fMRI data of six patients with focal epilepsy who underwent multimodal assessment using simultaneous EEG/fMRI acquisition followed by surgery. Only patient with a seizure free outcome were selected and the resected area was identified using a post-operative MRI. DANI automatically detected abnormal changes in 5 out of 6 patients, with excellent sensitivity, showing for each of them at least one “abnormal” lateralized network closely related to the epileptic focus. For each patient, we also detected some distant networks as abnormal, suggesting some remote reorganization in the epileptic brain.

## Introduction

Connectivity analysis in resting-state functional magnetic resonance imaging (rs-fMRI) is a promising tool to study neurological disorders. So far, rs-fMRI has been applied mainly at the level of groups of patients, as for instance in mental disorders (Broyd et al., 2009), in Alzheimer's disease (Goveas et al., 2011; Damoiseaux et al., 2012; Jacobs et al., 2013) and also in epilepsy (Bernhardt et al., 2013; Constable et al., 2013; Lang et al., 2014). However, a large amount of inter-patient variability is typically observed in any neurological disorder. In some applications, patient-specific features are the only clinically useful information. A prominent example is the multimodal investigation of patient with drug-resistant epilepsy, which aims at identifying an epileptogenic focus that could be surgically resected (Stefan et al., 2011; De Ciantis and Lemieux, 2013). The main goal of this study is to develop a method to capture inter-individual variations in resting-state networks (RSNs), and assess its potential usefulness in patients with focal epilepsy.

Usually, the analysis of patient-specific epileptogenic focus is based on analysing brain activity at the time of epileptic discharges. However, epileptic discharges are spontaneous and rare events that may not occur during time-limited standard neuroimaging investigations, such as simultaneous electro-encephalography/functional magnetic resonance imaging (EEG/fMRI) (Gotman and Pittau, 2011) or magneto-encephalography (MEG) (Stefan et al., 2011) explorations. When studying resting-state activity in the absence of epileptic discharges, some group-level studies have demonstrated rs-fMRI connectivity patterns specific to idiopathic generalized epilepsy (IGE) (Luo et al., 2011; Maneshi et al., 2012) and temporal lobe epilepsy (TLE) (Waites et al., 2006; Liao et al., 2011; Pittau et al., 2012b). For example, using seed regions in the epileptic focus, group comparison between TLE patients and age-matched healthy controls, we found significant decreases in connectivity between homologous mesio-temporal structures and also with the dopaminergic mesolimbic and with the default mode network (Pittau et al., 2012b). For IGE patients, we found significant increases and decreases in FC when using seeds in the attention network (Maneshi et al., 2012). The few studies, which have investigated FC changes at the individual level in patients with epilepsy, did report some reorganization of functional networks, notably in terms of their laterality (Negishi et al., 2011; Stufflebeam et al., 2011; Luo et al., 2014). Taken together, these results support our main hypothesis that resting-state fMRI connectivity could identify clinically relevant information in individual patients suffering from epilepsy.

Resting-state functional connectivity captures the spatial correlations of spontaneous fluctuations in the blood-oxygen-level dependent (BOLD) fMRI signal (Biswal et al., 1995). Maps of functional connectivity, i.e., temporal correlations of fMRI time series across brain regions, reveal highly organized spatial RSNs. These maps were found to be reproducible at the individual (Himberg et al., 2004; Shehzad et al., 2009) and at the group levels (Damoiseaux et al., 2006). Each RSN is a combination of multiple brain regions, not necessarily spatially contiguous, which share similar low frequency BOLD signal fluctuations (Fox and Raichle, 2007). These networks capture some aspects of the functional organization of the brain (Yeo et al., 2011). Many methods have been proposed to identify RSNs, including mainly variants of independent component analysis (ICA) (Smith, 2012) and cluster analysis (Yeo et al., 2011), see Smith et al. (2013) for a review. Initial applications of these techniques have focussed on group level analysis, (e.g., Damoiseaux et al., 2006), and it is only recently that their capacity to establish a correspondence between group and individual RSNs has been a topic of active research. Techniques available to address this problem include back-reconstruction in ICA (Calhoun et al., 2009) and dual-regression ICA (Beckmann et al., 2009). Here, we decided to build on a technique called Bootstrap Analysis of Stable Clusters (BASC) (Bellec et al., 2010), because of two of its unique features. First, the technique offers a statistical framework to assess the stability of RSNs at the individual and at the group level, by replicating a cluster analysis many times after small perturbations of the original dataset. This quantification of stability is an asset to establish whether atypical RSN organization observed in an individual can simply be attributed to statistical noise or reflects biological individual characteristics. Second, BASC explicitly looks at the modular organization of the brain, i.e., the ability to identify clusters based on the relative strength of intra-network vs. inter-network connectivity, independently of the absolute value of connectivity measures (see Alexander-Bloch et al., 2012 for a discussion of the distinction between connectivity and modularity). Dual-regression and back-reconstruction ICA, by contrast, perform a regression of temporal dynamics, which is sensitive to the absolute magnitude of connectivity. Absolute measures of functional connectivity are particularly sensitive to physiological noise, in particular motion (Power et al., 2012). We hypothesized that modularity would be more robust to physiological noise than absolute measures of connectivity.

To the best of our knowledge, none of the standard data-driven techniques for RSN mapping (ICA, clustering) has been evaluated at the individual level. They have rather been used to detect changes between two groups. The objective of this study was thus to develop and validate a statistical methodology, entitled “Detection of Abnormal resting state Networks in Individuals,” (DANI) aiming at identifying RSNs with atypical, or outlier, spatial distribution, when compared to a population of controls. The outlier RSNs were characterized by differences in stability and spatial extent with respect to a typical RSN distribution. We also extended BASC to include the notion of core of stability, defined as the most stable regions of a network. Because every RSN includes regions with fairly unstable cluster assignment, we hypothesized that focussing on RSN cores rather than on full networks would translate into improved characterisation of individual biological variability. In the first part of the paper, we assessed the ability of DANI to identify atypical and individual modular organization on a battery of simulated datasets.

Evaluation of RSN mapping for real individual fMRI datasets is challenging because of the lack of ground truth. The quality of individual mapping has been mainly assessed by test-retest reliability studies (Shehzad et al., 2009; Zuo et al., 2010) but test-retest reliability in itself does not indicate if reliable features are clinically meaningful. Concerning patients with epilepsy, the seizure outcome after surgery remains the gold standard to validate a technique. For this reason, in the second part of this study, DANI was applied to resting state fMRI data of six patients with focal epilepsy who underwent multimodal assessment using simultaneous EEG/fMRI acquisition followed by surgery.

## Materials and methods

### Subject selection

We selected healthy control subjects who underwent simultaneous EEG/fMRI acquisitions (Gotman et al., 2004), with the following inclusion criteria:

- Right-handed.
- EEG/fMRI runs during which the subject was awake: EEG stage W according to Iber and American Academy of Sleep Medicine (2007).
- EEG/fMRI runs involving only minimal motion (less than 1 mm translation and 1° rotation between volumes).

Based on these criteria, we selected 25 right-handed healthy control subjects The mean age was 32.8 years, ranging from 18 to 55. Written informed consent was obtained according to the guidelines and approval of the Montreal Neurological Institute research ethics review board. Note that this database of healthy controls acquired in our laboratory was the same as the one considered in our previous study (Pittau et al., 2012b).

### Simultaneous EEG/fMRI acquisition

EEG was continuously recorded as described in Gotman et al. (2004) inside a 3T MRI scanner (Siemens, Trio, Germany). The EEG acquisition was performed with 25 MR compatible electrodes (Ag/AgCl) placed on the scalp using the 10–20 (reference at FCz) and the 10–10 (F9, T9, P9, F10, T10, and P10) placement systems. Two electrodes were placed on the back to record the electrocardiogram. The head of the subject was immobilized with a pillow filled with foam microspheres (Siemens, Germany) to minimize movement artifacts and for subject's comfort. Data were transmitted from a BrainAmp amplifier (Brain Products, Munich, Germany, 5 kHz sampling rate) to the EEG monitor located outside the scanner room via an optical fiber cable.

A T1-weighted anatomic acquisition was first done (1 mm slice thickness, 256 × 256 matrix; echo time *TE* = 7.4 ms and repetition time *TR* = 23 ms; flip angle 30°). This scan was used for co-registration purposes and to superimpose the functional images on the anatomy. Functional data were acquired in runs of 6 min using a T2^*^-weighted EPI sequence (64 × 64 matrix; 33 slices, 3.7 × 3.7 × 3.7 mm, *TE* = 25 ms, *TR* = 1.9 s; flip angle 90°). Subjects were instructed not to move and stay with eyes closed, resting. Three runs were selected during wakefulness, with less than 1 mm of variation between two volumes for the three axes in translation and less then 1° of variation between two volumes for the three axes in rotation.

### fMRI data preprocessing

The fMRI database was preprocessed using the Neuroimaging Analysis Kit (NIAK) release 0.7^1^ (Bellec et al., 2011). Each fMRI dataset was corrected for inter-slice difference in acquisition time. Parameters of a rigid-body motion transformation were estimated for each time frame. Rigid-body motion was estimated within as well as between runs. The median volume of one selected fMRI run for each subject was coregistered with the individual anatomical T1 scan with Minctracc (Collins and Evans, 1997), using a rigid transformation. The T1 MRI of each subject was itself non-linearly co-registered to the Montreal Neurological Institute (MNI) stereotaxic template (Fonov et al., 2011), using CIVET pipeline (Zijdenbos et al., 2002). We used the MNI symmetric template, generated from the ICBM152 sample of 152 young adults, after 40 iterations of non-linear co-registration. The rigid-body fMRI-to-T1 transform and the non-linear T1-to-stereotaxic transform were all combined, and the functional volumes were resampled in the MNI space at a 3 mm isotropic resolution. The “scrubbing” method proposed by Power et al. (2012) was used to remove the volumes with excessive motion, i.e., all frames showing a displacement greater than 0.5 mm were removed. On average, 4% of the frames were thus removed using this “scrubbing” method. The following nuisance parameters were regressed out from the time series at each voxel: slow time drifts (basis of discrete cosines with a 0.01 Hz high-pass cut-off), average signals in conservative masks of the white matter and the lateral ventricles as well as the first principal components (accounting for 95% variance) of the six rigid-body motion parameters and their squares (Lund et al., 2006; Giove et al., 2009). The fMRI volumes were finally spatially smoothed with a 6 mm isotropic Gaussian blurring kernel. A more detailed description of the pipeline can be found on NIAK website (http://www.nitrc.org/projects/niak/).

To reduce the computational burden of the analysis, the spatial dimension of the individual fMRI dataset was reduced using a region-growing algorithm. The spatial dimension was selected arbitrarily by setting the maximal size where the growing process stopped: we chose a threshold of 800 mm^3^ resulting in *R* = 739 regions. The regions were built to maximize the homogeneity of the time series within the regions, i.e., the average correlation between the time series associated with any pair of voxels of the region. The region growing was applied on the time series concatenated across all subjects (after transformation to zero mean and unit variance), such that the homogeneity was maximized on average for all subjects, ensuring the use of small homogeneous and identical regions for all subjects. Because of the temporal concatenation of time series, we had to limit the memory demand, and the region-growing was thus applied sequentially and independently within each of the 116 anatomical areas of the AAL atlas (Tzourio-Mazoyer et al., 2002). See Bellec et al. (2006) for evaluation and further details regarding the implementation of this region-growing algorithm. Overall, this process reduced the dataset *Y* of each subject into a (*T* × *R*) data array, where *T* is the number of time samples and *R* is the number of regions.

The analysis of neuroimaging databases typically involves a large number of inter-connected steps. We used the Pipeline System for Octave and Matlab PSOM (Bellec et al., 2012) to execute processes in parallel on a cluster of workstations.

### Full brain functional connectivity analysis using BASC

Starting from preprocessed resting state fMRI data, we used the clustering method entitled BASC (Bellec et al., 2010) to quantify FC patterns at the individual and at the group level. BASC models FC between distant regions using spatial clustering of BOLD time courses. The key idea of BASC is to associate spatial clustering with Bootstrap resampling (Efron and Tibshirani, 1993) to assess the stability of such clustering among several replications, thus leading to a statistical measure of stability of the FC patterns. Since all the analyses were first done at the individual level and then across subjects at a group level, BASC offers a unique possibility to compare individual-level and group-level identifications of RSNs.

#### BASC analysis at the individual level

For each data matrix *Y* obtained at the individual level, FC was quantified using a spatial k-means clustering. Each clustering estimated an *R* × *R* binary adjacency matrix, setting a value of 1 when two regions were associated to the same cluster, and 0 otherwise. To assess the statistical stability of this clustering, the data matrix *Y* (*T* × *R*) was resampled using circular block bootstrap of the BOLD time-series, and a *k*-means clustering was then applied on each of the *B* = 300 replications. The average of all the *B* adjacency matrices resulted in a stability matrix $\hat{I}$_*i*_ (*i* = 1, …, *C*, being the subject index) representing the likelihood to cluster together the time-series of two regions.

#### BASC analysis at the group level

A similar process was considered to assess FC patterns at the inter-subject group level. To do so, the individual stability matrices of all the subjects $\hat{I}$_*i*_ (*i* = 1, …, *C*) were first averaged in order to generate a first group stability matrix. In a second step, a hierarchical clustering was applied using the Euclidean distance between columns of this group stability matrix as a similarity index to identify the columns with similar stability profiles. A threshold was then applied to cut the tree at L number of clusters, thus providing a binarised group level adjacency matrix (matrix exhibiting a 1 when two regions were associated within the same cluster, 0 otherwise). In order to take into account the statistical stability of such a clustering at the group level, standard bootstrap resampling among the C subjects was applied 1000 times and the same hierarchical clustering procedure was applied on each bootstrap sample.

By averaging the resulting 1000 binary adjacency matrices at the group level, we obtained the stability matrix at the group level $\hat{G}$ representing the likelihood to cluster together pairs of regions, while taking into account both single subject and group inherent variability of the data.

### Partition of the brain into functional RSN

To identify Consistent Resting State Networks (CRSNs) at the group level, the stability matrix $\hat{G}$ was converted into a partition of the whole brain, grouping regions that have been frequently associated within the same cluster, i.e., regions exhibiting high FC stability in $\hat{G}$ through bootstrap resampling. To do so, a last hierarchical clustering was applied on $\hat{G}$ to identify brain regions depicting similar stability profiles. Here again, the Euclidean distance between columns of $\hat{G}$ was used as a similarity index to identify the columns with similar stability profiles. A threshold was then applied to cut the tree at the desired number of clusters. We decided to threshold the dendrogram in *N* = 12 clusters, thus ensuring that we did not miss any important CRSN. Indeed, the literature usually refers to 7–10 CRSNs in healthy subjects (Damoiseaux et al., 2006; Smith et al., 2009). Note that for *N* = 12 CRSNs, we estimated *k* = 13 for the individual level *k*-means clustering and *L* = 14 to threshold each group level hierarchical clustering. These thresholds were estimated by optimizing a stability contrast as proposed in Bellec et al. (2010). This is a two-pass procedure, a first pass consisted in a fast (*B* = 30 bootstrap samples) exploration of a large grid of scales to find the scales (k(N), L(N), N) maximizing the stability of the clustering, as measured with a modified silhouette criterion of the group stability matrix, constraining *k* and *L* values within a close neighborhood of *N*. Local maxima of the modified silhouette (as a function of *N*) were then automatically identified. We chose to focus here on the local maximum found for *N* = 12, as this level of RSN decomposition was most similar to the ones traditionally reported in the literature (Damoiseaux et al., 2006). For such a scale of *N* = 12, the optimal parameters of *k* = 13 and *L* = 14 have been estimated. Following the scale selection procedure, the individual stability matrices for *N* = 12 were estimated a second time with a larger number of bootstrap samples (*B* = 300).

**Figure 1:**
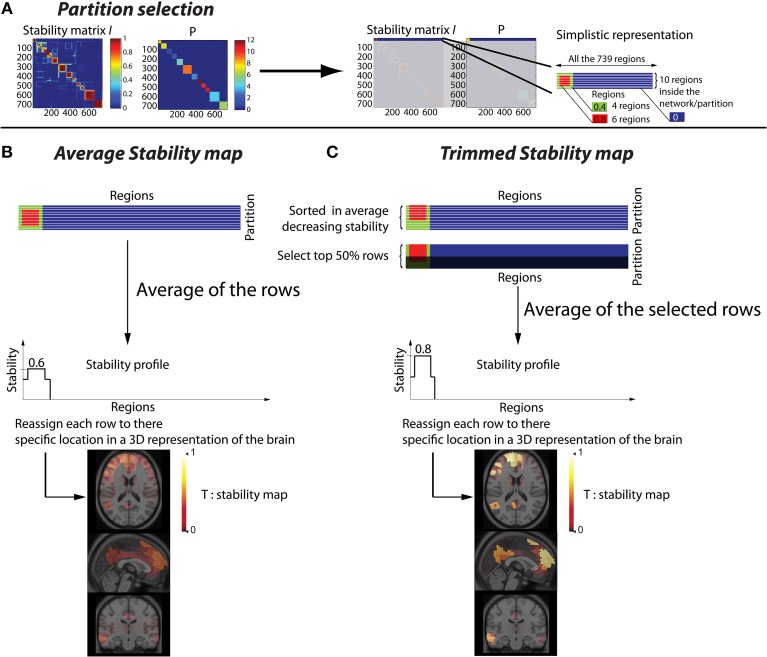
**Methodology to convert a stability matrix into a spatial stability map for each network.** The same process is applied for each cluster of the partition *P*, thus every stability map was constrained by the corresponding CRSN. **(A)** Simplistic representation in which the first cluster of the partition *P* was selected on the stability matrix. **(B)** Averaged stability map obtained by averaging all the rows inside a network partition, resulting in a diffuse and low contrast representation of the network. **(C)** Trimmed stability map obtained by averaging the 50% rows showing the largest stability values, resulting in a representation of the network with greater contrast.

Let us define as *P*(*r*) for *r* ∈ [1, *R*] a vector representing the resulting partition obtained after thresholding the hierarchical clustering of $\hat{G}$ in *N* clusters. When two regions are associated within the same cluster, they are associated to the same label in *P* (Figure 1A).

### Detection of abnormal networks in individuals (DANI)

BASC allows estimating stability of FC patterns at the individual level and at the group level, what we will call modules. The objective of DANI is to detect, at the individual level, possible modifications of those modules that could be considered as deviant or outliers when compared to a population of controls.

#### BASC extension: from stability matrices to trimmed stability maps

Starting from the stability matrices estimated at the individual level $\hat{I}$_*i*_ or at the group level $\hat{G}$ using BASC, our first contribution was to propose a method to improve the visualization of spatially FC information represented in these stability matrices. Therefore, we propose to convert a stability matrix into a series of *N* **stability maps**. Each of these maps is generated using information provided by one of the *N* clusters of the partition *P*. Consequently, the stability maps are reporting for each region the amount of stability estimated for one particular cluster of the partition. We will refer to each of these spatial maps as a network.

This procedure to estimate “**trimmed stability maps**” is applied on each cluster of the partition. It aims at generating 3D stability maps with enhanced contrast and increased consistency across runs. To do so, the rows of each cluster of the partition matrix *P* were first reordered in decreasing order of average stability. We then considered a percentage μ of most stable rows, representing the stability cores. The stability profile was then estimated by averaging only these “most stable rows.” The resulting *3D trimmed stability map* obtained from this procedure is illustrated in Figure 1C. It yielded greater contrast than when simply averaging all the rows of the cluster (Figure 1B). We performed simulations varying the values of the parameter μ of 25, 50, and 75% to assess the impact of such threshold on the sensitivity of our detection method. It is important to mention that, whereas only rows corresponding to a particular cluster of *P* are selected, stability values from all *R* regions are actually averaged to generate stability maps. Consequently regions not belonging to that specific cluster may also exhibit some non-null stability values.

The 3D trimmed stability maps estimated at the group level from the stability matrix $\hat{G}$ represent the CRSNs. These CRSNs were considered as our reference when characterizing the functional organization of RS brain activity over a population of healthy controls.

The 3D trimmed stability maps, estimated at the individual level from the stability matrices $\hat{I}$_*i*_, characterized the amount of stability assessed for each subject, within each of the CRSNs identified at the group level. Since all maps were estimated within the same referential space, comparison between maps estimated at the individual level and at the group level became feasible. Estimating all trimmed stability maps from the same partition is a strong constraint but it was necessary to provide consistency across subjects. While providing the similar basis for comparison, this method allowed flexibility to adapt to the particularities of each individual.

#### Detection of abnormal modifications of functional connectivity

Let us define as *CI*^*c*^_*n*_ (*r*) the *R* × *N* × *C* matrix containing the trimmed stability values of the region *r* (*r* = 1 … *R*) for the network *n* (*n* = 1… *N*) and for the healthy control subject *c* (*c* = 1 … *C*). For a specific target subject to be tested with DANI, let us denote *T*_*n*_ the vector of size *R* containing the trimmed stability values for the network *n* (*n* = 1… *N*) for this particular subject. The objective of DANI is to identify automatically which of the trimmed stability maps *T*_*n*_ could be considered as outliers when compared to the trimmed stability maps of all controls (*CI*^*c*^_*n*_, *c* = 1 … *C*). The first step was to detect networks exhibiting variations in stability when compared to controls maps. The second step was to quantify whether these variations were statistically significant.

***Detecting stability variations in the functional network organization***. When performing a region-based comparison of an individual map *T*_*n*_ with all *CI*^*c*^_*n*_ maps (*c* = 1 … *C*) from a population of controls, it was not possible to use a standard *Z*-score. Indeed, each map quantifies stability in FC within a network, i.e., estimated from a predefined cluster of the partition *P*. Consequently these maps are not continuous through the whole brain volume (see Figure 1C). Whereas some regions outside the corresponding cluster may exhibit non-null stability values, many other voxels exhibit stability values very close or equal to zero. Consequently, estimating a voxel-based mean and a standard deviation both close to zero among the controls would lead to unstable *Z*-score values. Indeed, one can obtain large *Z*-values, even though the local stability was very close to zero. This issue can cause problems of interpretation since it will attract attention to a non-stable area of rare occurrence.

To address this issue, instead of computing *Z*-scores, we propose to use a binary mask, denoted *Zmask*_*n*_, to highlight brain regions depicting significant differences in stability for the target subject and the network *n* when compared to the population of *C* controls. These masks were defined as follows:

(1) $$Zmask_{n}\left(r\right)=\left[\left|T_{n}\left(r\right)-\bar{CI_{n}\left(r\right)}\right|>3.17\;\times std\left(CI_{n}\left(r\right)\right)\right]$$

Where *r* denotes the region within the brain volume (*r* = 1 … *R*), *CI*_*n*_(*r*) and *std* (*CI*_*n*_ (*r*)) refer to the mean and the standard deviation maps estimated over all *CI*^*c*^_*n*_ maps (*c* = 1 … *C*), for the network *n* (*n* = 1 … *N*). 3.17 was chosen as the *Z*-threshold considered for a non-corrected significant level of *p* < 0.001. Note that *Zmask*_*n*_ will identify any significant change in the target subject, whether it consisted in an increase or a decrease of stability.

***Combined map assessing the amount of stability within the detected Zmask_n_***. Since stability values estimated using BASC are statistical measurements, i.e., a probability to belong to a specific network, we created a map exhibiting these stability values within brain regions showing significant differences between the target subject and the population of controls. To do so, we applied the *Zmask*_*n*_ to the difference between the individual subject-map *T*_*n*_ and the mean stability map *CI*_*n*_ (*r*) from all the controls (see Figure S1).

(2) $$Cmap_{n}\left(r\right)=Zmask_{n}\left(r\right)\times \left[T_{n}\left(r\right)-\bar{CI_{n}\left(r\right)}\right]$$

**Figure 2:**
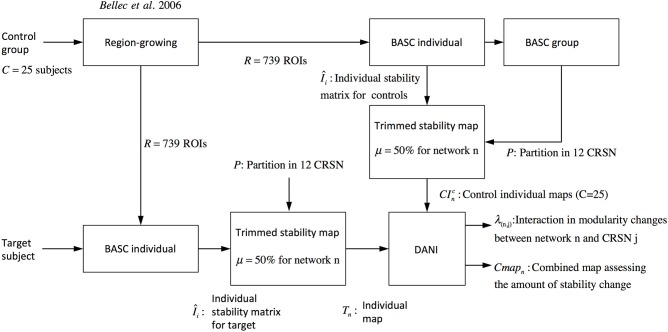
**Pipeline summarizing the steps involved in DANI method starting from the preprocessed fMRI images of the control population and the preprocessed images of the target subject on which DANI was applied**.

Where *r* denotes the region within the brain volume. Using *Cmap*_*n*_ instead of *Z*-scores maps will avoid biasing the interpretation toward brain regions showing large *Z*-score values and very low stability values. Note that Figure 2 is showing a graphical summary of all the steps included in this pipeline.

#### Thresholding the combined map *Cmap_n_*

In order to identify outliers when investigating changes in stability for a particular target subject, the null hypothesis distribution of *Cmap*_*n*_ values was assessed by applying Equation (2) on each of the *C* subjects of the control database and for each of the *N* networks. Therefore, we estimated the distribution of so-called “stability changes” likely to occur within a healthy control population. When pooling values from all *C* = 25 controls and *N* = 12 networks, we obtained 0.1 and 99.9 percentile values of −0.34 and 0.34, respectively. Therefore, in order to consider only stability changes likely to be the most significant at the individual level, all *Cmap*_*n*_ values between −0.34 and 0.34 were threshold and subsequently set to zero for the next analyses.

#### Analysis of the most salient findings for a specific individual

As mentioned earlier, stability measurements considered in this study constitute already statistical measurements. Therefore, we propose to consider first the most salient findings identified for every target subject, i.e., for every patient with epilepsy selected for this particular study. Consequently, for each of the six selected patients, we carefully inspected all the *N* = 12 *Cmap*_*n*_ and only the maps exhibiting a maximum stability increase greater than 0.5 or a minimum stability decrease lower than −0.5 were reported. Since the 0.1 and 99.9 percentiles of stability changes measured over the control database were, respectively −0.34 and 0.34, the threshold of 0.5 was chosen arbitrarily in order to investigate only the most salient findings.

#### Automatic detection of modularity changes for a specific individual

In addition to the inspection of all *Cmap*_*n*_ results, we also propose a statistical analysis to automatically detect significant changes in modularity. To do so, we proposed a new metric assessing, for each individual network of each target subject, the strength and spatial extent of stability interaction when compared to other CRSNs.

Let us define *W*_*n*_ (*r*) a 3D spatial map estimating which of the *N* CRSNs defined in the partition *P* (*r*) were involved in local modifications detected using *Zmask*_*n*_ (*r*):

(3) $$W_{n}\left(r\right)=P\left(r\right)\times Zmask_{n}\left(r\right)$$

This operation simply consists in applying the binary mask *Zmask*_*n*_ to the partition *P*. We then introduce the metric λ_*n,j*_, for *n* = 1 … *N* and *j* = 1 … *N*, assessing the interaction between local changes of stability of the *n*th individual network (*Cmap*_*n*_) with the *j*th CRSN (i.e., voxels where (*W*_*n*_ (*r*) == *j*)):

(4) $$\lambda_{n,j}=\sum_{r}\left(\left(W_{n}\left(r\right)==j\right)\times \left|Cmap_{n}\left(r\right)\right|\right)$$

λ_*n,j*_ quantifies the amount of stability change with which the *j*th CRSN contributes to the *n*th network of the target subject to be evaluated. As a summation, this metric is sensitive to both the strength and the spatial extent of the stability changes. It is important to mention that this metric is estimated only within *Zmask*_*n*_, therefore only significant changes in stability are accounted for and only within regions belonging to the *j*th CRSN.

Therefore the *N* × *N* matrix λ_*n,j*_ provides an overview of all local changes in FC of a target subject when compared to all *N* CRSNs extracted from the control database.

Using the interaction metric λ_*n,j*_, outlier detection was applied in order to detect abnormal networks, i.e., interactions that are very unlikely to occur for a specific subject. Since the a priori null distribution of this metric is not known, a non-parametric test was considered. To estimate the null hypothesis distribution, a *N* × *N* matrix λ_*n,j*_ was first estimated for each of the *C* subjects of the control database. The null distribution for each network n and CRSN j was then estimated using a generalized jackknife approach (Sharot, 1976). To do so, 2/3rd of the control sample was randomly selected to calculate the mean and standard deviation and one target sample was randomly selected among the remaining 1/3rd to compute the metric λ_*n,j*_ under the null hypothesis. The procedure was perform 10,000 times using the *C* = 25 controls to characterize the null distribution H0 for each network *n* and CRSN *j*. The values of the metric λ_*n,j*_ estimated for the target subject were then compared to the corresponding H0 distribution and *p*-values were estimated.

### Validation of DANI using realistic simulated data

The objective of this section is to further evaluate DANI using simulated data obtained within a fully controlled realistic environment, thus providing a gold-standard to assess the performance of the method.

#### Generation of simulated data

We evaluated the performance of DANI by adding different levels of structured signal to perturb the underlying “network” organization of real resting state fMRI state data. This structured signal actually consisted in the average time series of all regions belonging to the visual network (CRSN #4) of another control subject. We also evaluated the influence, on DANI detection properties, of the core size parameter μ considered when estimating the trimmed stability map.

The simulated Signal-to-Noise Ratio (SNR) was defined as follows:

(5) $$SNR_{dB}=20log_{10}\left(\frac{RMS_{data}}{RMS_{simul}}\right)$$

Where *RMS* is the root mean square amplitude of the time-series corresponding to the region where the perturbation was applied. We refer to *RMS*_*data*_ as the root mean square of the original fMRI time-series of the subject and as *RMS*_*simul*_ as the root mean square of the time-series introduced to perturb the system.

***Perturbation with structured signal***. To force the fusion of two networks and assess the ability of DANI to detect it, structured signal was added on specific parts of two networks within resting state fMRI data of one control subject. Regions belonging to parts of the right hemisphere of the sensory-motor network (CRSN #9) and the auditory network (CRSN #3) were selected as the area to be perturbed (denoted area A). Structured signal was estimated as the average time series of all regions belonging to the visual network (CRSN #4) of another control subject. We then assessed, at what *SNR*_*dB*_ level, DANI could detect the fusion of these two parts of the perturbed networks, varying *SNR*_*dB*_ from −25 to 25 dB by steps of 1 dB. Small *SNR*_*dB*_ values then corresponded to the addition of a large amount of structured signal to corrupt an area involving some regions of the sensory-motor and auditory networks (Figure S2).

#### Validation metric

To quantify the performance of DANI when applied on these simulated data, we first estimated the trimmed stability map of the sensory-motor (**Figure 4**, CRSN #9) and the auditory (**Figure 4**, CRSN #3) networks, using a specific core size parameter μ. For each of these two CRSNs, we evaluated the resulting estimated stability values inside and outside the perturbed zone on which structured signal was added, but limited to the boundaries of the CRSN of interest. Let us define as *I*_*n*_0__ the average of all stability values inside the perturbed zone A for a specific network *n*_0_.

(6) $$I_{n_{0}}=\;\frac{1}{card\left(A\right)}\sum_{r\in A}T_{n_{0}}\left(r\right)$$

Where *card* (*A*) refers to the number of voxels belonging to the perturbed zone A and *T*_*n*_0__ is the trimmed stability map of the target subject for the network *n*_0_.

Let us define as *A*′ the area of the specific CRSN *n*_0_ located outside the perturbed zone, we introduced the metric *O*_*n*_0__ as the average of all the stability values outside the perturbed zone *A* but within a specific network *n*_0_.

(7) $$O_{n_{0}}=\;\frac{1}{card\left(A^{\prime }\right)}\sum_{r\;\in \;A^{\prime }}T_{n_{0}}\left(r\right)$$

**Figure 3:**
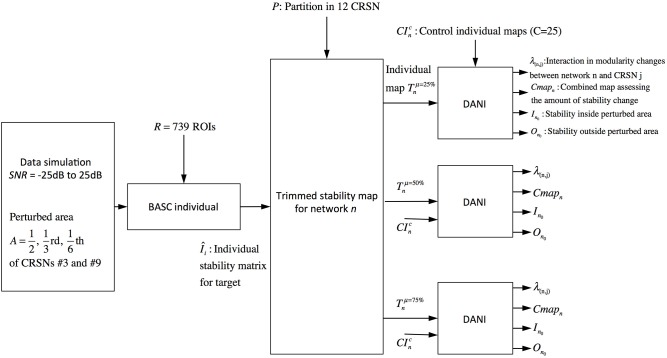
**Pipeline summarizing the validation procedure: the input is the preprocessed fMRI data perturbed within a region of size A (A = 1/2, 1/3, and 1/6 of the targeted networks) at a specific *SNR*_*dB*_ and the outputs are the metrics *Cmap*_*n*_, λ_*n,j*_, *I*_*n*0_ and *O*_*n*0_.** Simulations were performed considering 3 core sizes (μ = 25, 50, and 75%) for the estimation of the trimmed stability maps.

The validation metrics *I*_*n*_0__ and *O*_*n*_0__ were evaluated for different *SNR*_*dB*_ levels over two different perturbed networks, namely the sensory motor network and the auditory network. Figure 3 presents a schematic overview of this validation pipeline.

An increase in *I*_*n*_0__ would therefore be interpreted as the occurrence of a more stable and consistent network within the targeted region, whereas a decrease in *I*_*n*_0__ would be interpreted as a lost of the affected region in favor of another network. On the other hand, a decrease in *O*_*n*_0__ would mean that the outside of the original network is no longer associated with the new organization, whereas a stable *O*_*n*_0__ value would mean that the integrity of the original network was preserved.

#### Validation parameters

DANI performance was evaluated by varying the following parameters:

- The *SNR*_*dB*_ at which the structured signal was added ranged from -25 to 25 dB by steps of 1 dB.
- The size of the perturbed zone A varied from 1/2, 1/3rd, and 1/6th of the sensory-motor network and the auditory network.
- The core size parameter μ considered when estimating the trimmed stability maps varied from 25, 50, and 75% of the most stable rows in a given cluster.

### Evaluation of DANI on clinical data

This section describes the application of DANI on resting state fMRI data of patients with focal epilepsy, who underwent multimodal assessment using simultaneous EEG/fMRI acquisition followed by surgery.

#### Subject selection criteria for clinical evaluation

Among a population of patients with history suggestive of drug-resistant focal epilepsy (1989; Berg et al., 2010), we selected patients who underwent surgery after simultaneous EEG/fMRI investigation (Gotman et al., 2004), with at least 12 months follow-up. Candidates underwent routine presurgical evaluation, whereas EEG/fMRI was performed independently of other modalities and not considered for placing intracranial electrodes or for surgical decision.

Besides general criteria mentioned in Section Subject Selection, we included the following additional inclusion criteria:

- Seizure free patients with at least 12 months follow up. The location of the resection was obtained from postsurgical morphological MRI data.
- Patients who had at least two runs of EEG/fMRI showing epileptic discharges, for which the BOLD response to epileptic discharges was evaluated as either fully concordant or partially concordant with the location of the resection, following the methodology proposed in An et al. (2013).
- Patients who had also at least two runs of EEG/fMRI with no or small numbers of epileptic discharges on scalp EEG in order to investigate functional connectivity patterns independently from the occurrence of epileptic discharges.

Six patients were selected for this study: patients 1 and 2 had right orbito frontal epilepsy. Whereas the anatomical MRI was evaluated as non-lesional, a small focal cortical dysplasia (FCD) was confirmed by pathology. Patients 3 and 4 had, respectively left and right mesio-temporal lobe epilepsy (MTLE) with hippocampal sclerosis. Patients 5 and 6 had, respectively left and right frontal lobe epilepsy (FLE) with the presence of a FCD detected on the MRI and confirmed by pathology (see Table 1 for further details).

**Table 1:** **Patients' clinical data**.

**Numbers**	**Age/Gender**	**Anat MRI**	**Syndrome (Etiology)**	**Resection**	**Pathology**	**Follow up**
1	16/F	Normal	R FLE (NL)	R OF	FCD IIa	24
2	38/M	Normal	R FLE (NL)	R OF	FCD IIb	12
3	20/M	L MTS	L TLE (MTS)	L ant TL	Gliosis	12
4	39/F	R MTS	R TLE (MTS)	R ant TL	Neuronal loss and gliosis	24
5	26/F	L 2nd F gyrus FCD	L FLE (FCD)	L 2nd and 3rd F gyrus	FCD IIb	12
6	15/M	R F psagittal FCD	R FLE (FCD)	R 1st F gyrus	FCD IIb	36

R, Right; L, Left; FLE, Frontal Lobe Epilepsy; NL, Non-Lesional; OF, Orbito-Frontal; FCD, Focal Cortical Dysplasia; MTS, Mesio-Temporal Sclerosis; TLE, Temporal Lobe Epilepsy.

#### Estimation of the BOLD response to epileptic discharges

The analysis of BOLD response to epileptic discharges detected on scalp EEG was identical to the method considered in previous studies from our group (Bagshaw et al., 2004; Gotman and Pittau, 2011; An et al., 2013; Heers et al., 2014). fMRI data were preprocessed following a similar methodology than the one presented in Section fMRI Data Preprocessing. After correcting EEG data from MR gradient artifact (Allen et al., 2000) and ballistocardiogram artifact (Benar et al., 2003), EEG was reviewed by an expert epileptologist (FP) and epileptic discharges were marked. Timing and duration of each discharge were considered to generate regressors and convolved with four hemodynamic response functions (HRFs) peaking at 3, 5, 7, and 9 s, in order to model inherent variability of HRF in patients with epilepsy (Bagshaw et al., 2004). Motion parameters were modeled as confounds and all regressors were included in the same general linear model. A combined *t*-map was created by taking, at each voxel, the maximum t value from the four *t*-maps based on four HRFs. To be significant, a response required five contiguous voxels having a *t*-value > 3.17 (*p* < 0.05 using Bonferroni correction to take into account the four HRFs analyses).

#### Multimodal assessment

Postsurgical morphological MRI data consisted either in 3D high resolution T1 weighted MRI (1 mm isotropic resolution) or T2 weighted axial or coronal slices (in plane resolution: 0.46 mm, slice thickness: 5 mm). Postsurgical MRI data were co-registered to the high resolution anatomical MRI acquired during the EEG/fMRI session, by maximizing normalized mutual information (Studholme et al., 1999), assuming an affine geometrical transformation between the two volumes. Using the inverse transformations of the rigid-body fMRI-to-T1 transform and the non-linear T1-to-stereotaxic transform introduced in Section fMRI Data Preprocessing, DANI results, i.e., the combined maps of stability changes *Cmap*_*n*_ for all *n* = 1 … *N* networks, were resampled in the native space of the anatomical MRI of each patient. Therefore, DANI results, BOLD responses to epileptic discharges and postsurgical MRI data could be compared on a voxel/voxel basis with the native MRI space of every patient.

## Results

### Consistent resting state networks

**Figure 4:**
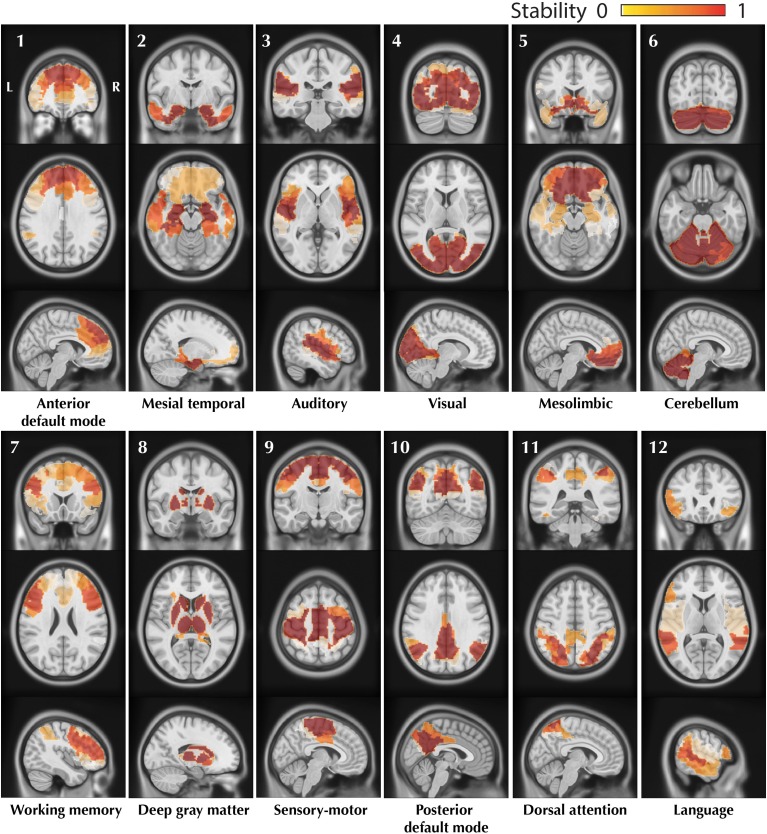
**Visual representation of the 12 CRSNs identified using BASC group level analysis of 25 healthy control subjects.** For each CRSN: 3 slices (coronal, axial, sagittal) are shown superimposed on an anatomical MRI template (MNI152). Labeling of each network was done visually based on previously reported CRSNs in the literature. The figure shows the usual networks: Default Mode Network (1,10), Auditory (3), Visual (4), Sensory-Motor (9), Attention (7,11) and Language(12). BASC also identified 4 other networks, less often reported, but characterized by high statistical stability: Mesio- Temporal (2), Mesolimbic (5), Cerebellum (6) and Deep Gray Matter (8).

The resulting trimmed stability maps obtained from group level BASC analysis of the 25 healthy controls, resulting in the identification of 12 CRSNs, are presented in Figure 4. These CRSNs were used as the reference functional networks to detect possible abnormal networks in patients.

### Validation of DANI using simulated data

**Figure 5:**
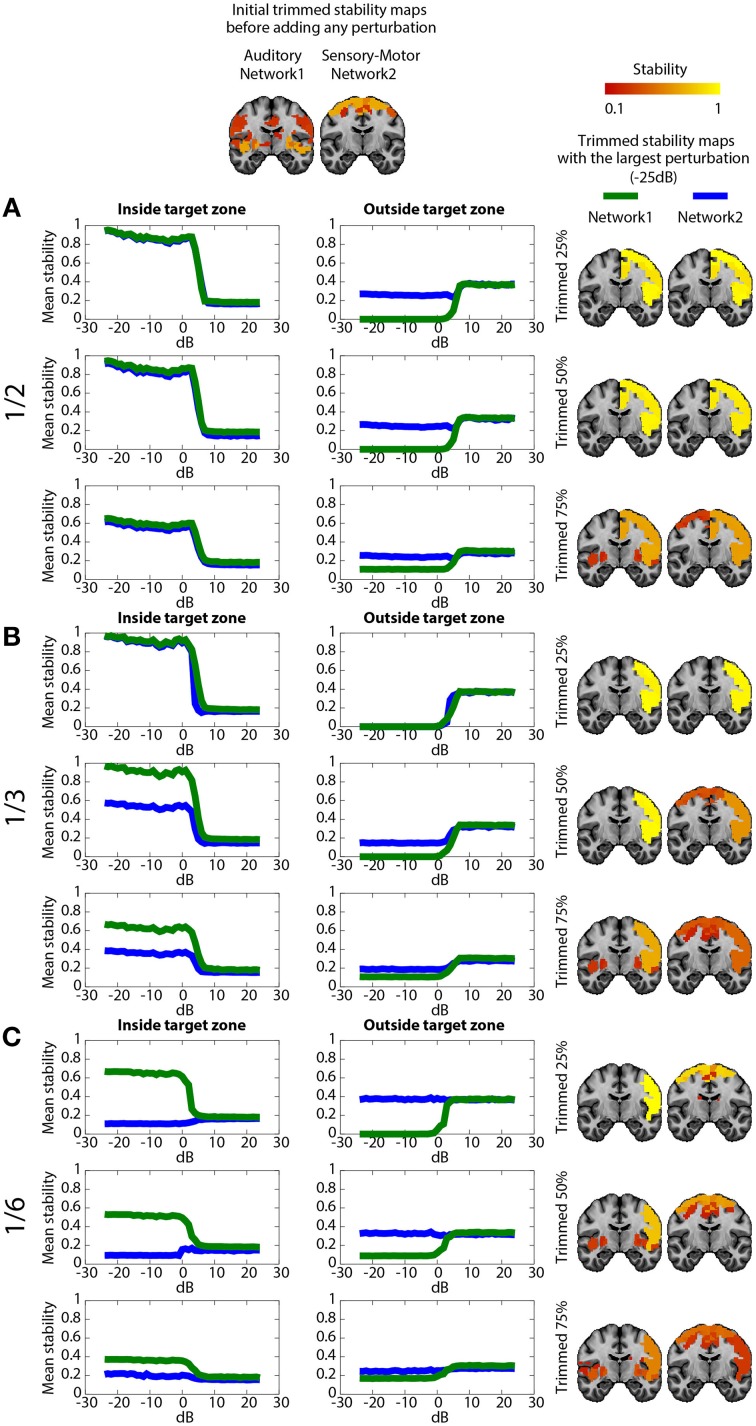
**Evaluation of DANI using simulated data.** The first line shows the initial trimmed stability maps *T*_*n*_ obtained for the Auditory network (network 1) and the Sensory-Motor network (network 2) before adding any perturbution. Graphs present the average stability inside *I*_*n*_0__ (left column) outside *O*_*n*_0__ (middle column) the perturbed area as a function of *SNR*_*dB*_ values ranging from −25 dB to +25 dB, for both the Auditory (green) and the Sensory-Motor (blue) networks. The right column shows the resulting trimmed stability maps *T*_*n*_ obtained when adding the largest perturbation at *SNR*_*dB*_ = −25*dB*. **(A)** perturbation zone involving 1/2 of both Auditory and Sensory-Motor networks, **(B)** perturbation zone involving 1/3rd of both Auditory and Sensory-Motor networks, **(C)** perturbation zone involving 1/6th of both Auditory and Sensory-Motor networks. In each case, results obtained when varying the core size parameter μ at 25, 50, and 75% are presented.

Figure 5 shows the impact on DANI results when adding structured signals to perturb resting state fMRI data from parts of the sensory-motor and the auditory CRSNs and when varying the core size parameter μ, i.e., the percentage of stability considered when estimating trimmed stability maps. The left column of the Figure represents the average stability inside the target zone as a function of *SNR*_*dB*_, the middle column shows the average stability estimated outside of the target zone as a function of *SNR*_*dB*_ and the third column shows the resulting trimmed stability maps for both networks at *SNR*_*dB*_ = −25 *dB*, i.e., at the highest perturbation level. Overall, DANI was able to identify changes around 7 dB regardless of the size of the target zone and with all core sizes. When adding perturbation in half (Figure 5A), 1/3rd (Figure 5B) and 1/6th (Figure 5C) of the two networks, choosing a core size μ of 25% drastically enhanced the stability within the target region (left column), whereas at the same time the resulting stability outside the target was significantly reduced, down to zero for the auditory network, meaning that this network was lost and completely taken over by its fusion with the sensory-motor network. Resulting trimmed stability maps obtained when most intense perturbation was added (*SNR*_*dB*_ = −25*dB*) are confirming this trend, since mainly the fused perturbed network was detected for both networks (Figure 5. right column). On the other hand, using a core size μ of 75%, only moderate changes could be detected from stability profiles especially when perturbation was added on 1/6th of the two networks (Figure 5C), yielding a poor stability contrast between regions of interest. This trend was also confirmed on the resulting trimmed stability maps obtained at *SNR*_*dB*_ = −25 *dB* (Figure 5 right column). Finally, choosing a core size μ of 50% provided a good trade-off yielding good sensitivity to detect the new fused network (Figure 5 left column), while providing accurate stability measures within the remaining sections of the non-perturbed networks (Figure 5 middle). This trade-off corresponding to an ideal detection contrast obtained at μ = 50% is illustrated on the resulted trimmed stability maps obtained at *SNR*_*dB*_ = −25 *dB* (Figure 5 right column).

When perturbing a large target zone involving half of both networks (Figure 5A), DANI detected one large fused network at the detriment of the two original ones. The remaining non-perturbed areas of the original networks were no longer classified as being part of the network and a new fused network was detected. We observed a different behavior when perturbing 1/3rd of the two networks (Figure 5B) instead of completely merging the two networks together, DANI partially merged them. When perturbing only a small region (1/6th of both networks, Figure 5C), the perturbed part of the sensory-motor network was associated with the auditory network, resulting in the detection of a smaller sensory-motor network and a larger auditory network.

### Evaluation of DANI on clinical data

Results from both statistical analyses applied on DANI clinical results are reported in Table 2. For every patient and for every network n, the maximum and minimum values of *Cmap*_*n*_ were first reported. Networks exhibiting salient stability increases, associated with a maximum value larger than 0.5, are indicated in red font. Note that no network exhibited salient stability decreases associated with a minimum value lower than −0.5. The second analysis consisted in an automatic detection of modularity changes using a non-parametric approach (see Section Automatic detection of modularity changes for a specific individual). Networks identified in a significant interaction, i.e., rejecting the null hypothesis λ_*n,j*_ = 0 with *p* < 0.001, were reported using a bold font and a “∗” sign in Table 2.

**Table 2:**
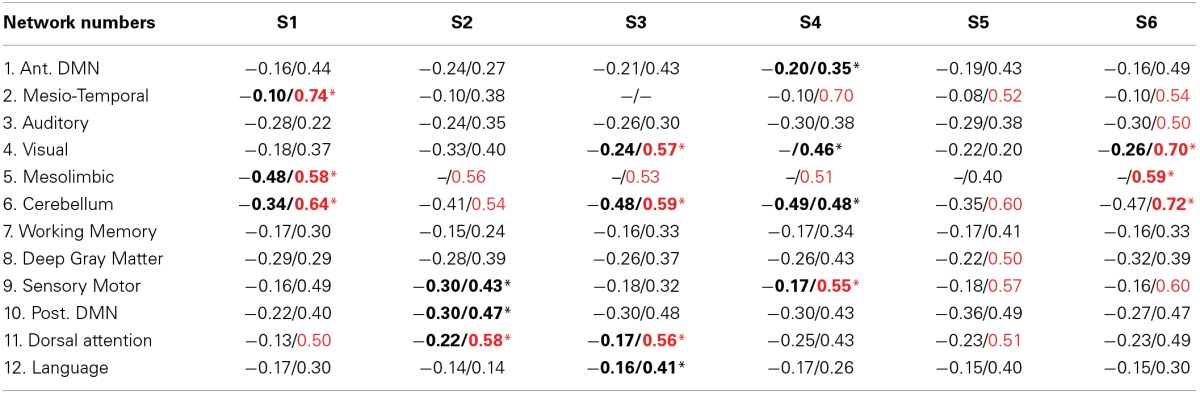
**Analysis of the most salient findings detected by DANI**.

For each of the 12 networks and for each patient, we are reporting the global maximum and minimum value of stability changes identified in *Cmap*_*n*_. Networks exhibiting the most salient increases in stability, i.e., more than 0.5, were indicated in red font (no salient findings showing a minimum decrease in stability lower than −0.5 were found). Network involved in significant interactions of modularity changes with other CRSN at p < 0.001 (cf. Section Automatic detection of modularity changes for a specific individual) were indicated with a “∗” and in bold font. (“-” indicates that no negative values were found in *Cmap*_*n*_).

Note that results for each patient are represented using *Cmap*_*n*_, measuring, for each network n, only the significant differences in stability observed for the target individual patient when compared to the population of controls. This is the reason why remote regions from the underlying CRSN partition could be detected in those maps, whereas main regions associated with the underlying CRSN partition might not be present if no significant stability changes were detected. All *Cmap*_*n*_ have been thresholded above the 99.9% percentile (resp. below the 0.1% percentile) measured over the control population, i.e., above 0.34 and below −0.34.

**Figure 6:**
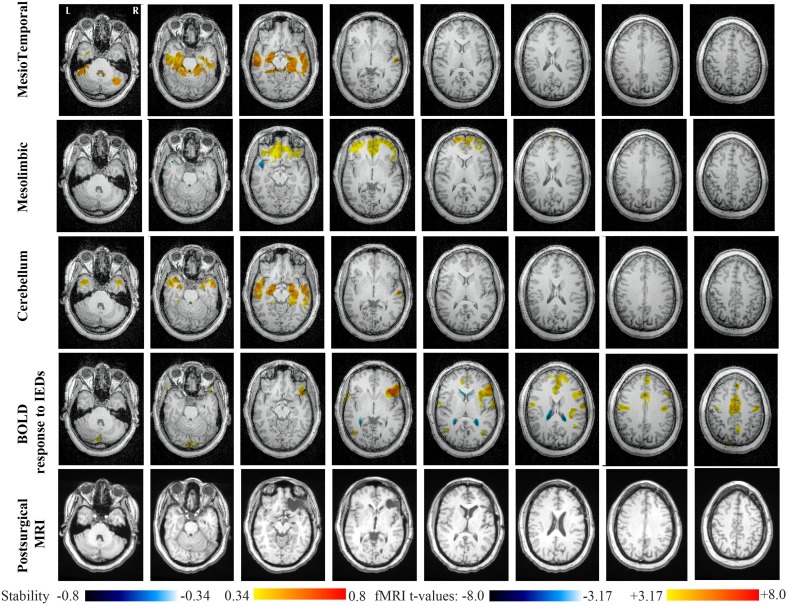
**Evaluation of DANI on Patient 1 with right Orbito-Frontal epilepsy.** Results are presenting most salient stability changes observed in *Cmap*_*n*_ followed by a t-map of the BOLD response to epileptic discharges and a postsurgical T1 MRI, all resampled in the native anatomical MRI space of the patient. Most salient stability changes observed for *Cmap*_*n*_ of Mesio-Temporal, Mesolimbic, and Cerebellum networks are presented. These three networks were also involved in significant interactions of modularity changes at *p* < 0.001. *Cmap*_*n*_ are presented between 0.34 and 0.8 (resp. −0.34 and −0.8) and BOLD *t*-map between 3.17 and 8.0 (resp. −3.17 and −8.0) using a yellow–red colormap (resp. white–blue colormap).

Main results obtained for patient 1 with right orbito-frontal epilepsy are presented in Figure 6. A BOLD activation to epileptic discharges was found in the right orbito-frontal focus and was fully concordant with the location of the resection (patient seizure free at 24 months). Whereas the anatomical MRI was evaluated as non-lesional, pathology confirmed a FCD within the focus. The networks that showed most salient stability increases in *Cmap*_*n*_ were the Mesio-Temporal, Mesolimbic, Cerebellum and Dorsal Attention networks. All these networks except the Dorsal Attention were also involved in significant interactions of modularity changes (*p* < 0.001). *Cmap*_*n*_ for the Mesolimbic network, containing the focus, showed increases in stability within the whole Mesolimbic network, involving notably the focus and showing increase in stability within most bilateral regions of this network. Increase stability suggest that, when compared to a population of controls, these regions are more reliably connected together for this specific patient. A local maximum in *Cmap*_*n*_ was found within the right frontal pole, actually in a close neighborhood around the focus. *Cmap*_*n*_ for the MesioTemporal network showed large increases in stability (up to 0.74, i.e., 74% more stable than within the control population) within bilateral mesial and lateral temporal regions and the Cerebellum. Connections between the Mesolimbic and Mesiotemporal networks are well-known propagation pathways in epilepsy. *Cmap*_*n*_ for the Cerebellum network showed increases in stability within bilateral mesial and lateral temporal regions, involving notably both temporal poles. *Cmap*_*n*_ for the Dorsal Attention network shows stability increases within itself, involving as well some regions of the posterior Default Mode Network (DMN) (results not shown).

**Figure 7:**
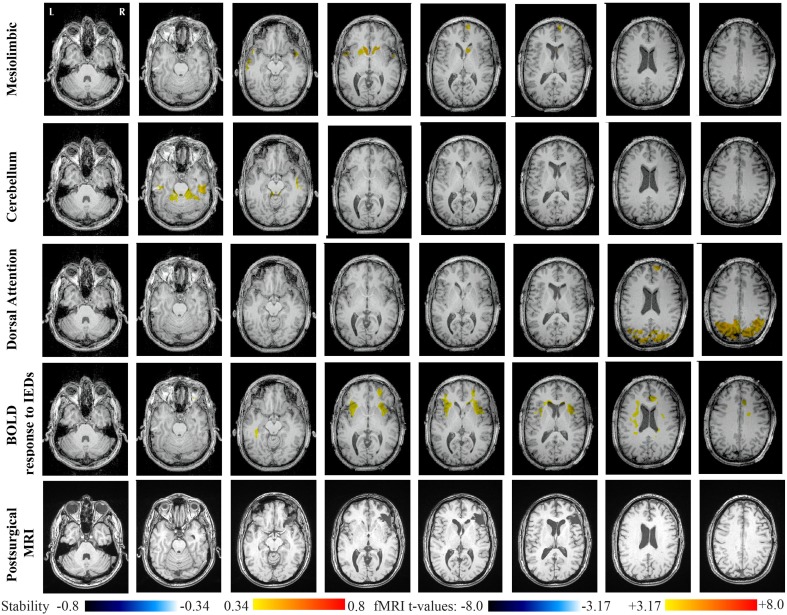
**Evaluation of DANI on Patient 2 with right Orbito-Frontal epilepsy.** Results are presenting most salient stability changes observed in *Cmap*_*n*_ followed by a t-map of the BOLD response to epileptic discharges and a postsurgical T1 MRI, all resampled in the native anatomical MRI space of the patient. Most salient stability changes observed for *Cmap*_*n*_ of Mesolimbic, Cerebellum, and Dorsal Attention networks are presented. The Dorsal Attention network was also involved in significant interactions of modularity changes at *p* < 0.001. Same colormap conventions than in Figure 6.

Main results obtained for patient 2 with right orbito-frontal epilepsy are presented in Figure 7. A BOLD activation fully concordant with the resected area in the right orbito-frontal region was also observed for this patient. The MRI was evaluated as non-lesional and a FCD was identified by pathology analysis. Most salient stability increases in *Cmap*_*n*_ were found for the Mesolimbic, Cerebellum, and the Dorsal Attention networks. On the other hand, the Sensori-Motor network, the posterior DMN and the Dorsal Attention network were also involved in significant interactions of modularity changes. *Cmap*_*n*_ for the Mesolimbic network showed stability increases within the right frontal pole (lateralized on the side of the focus), bilateral heads of the caudate nuclei and bilateral insulae regions, suggesting notably increase stability in several regions surrounding the focus. *Cmap*_*n*_ for the Cerebellum network showed stability increases in some regions of the Cerebellum, with some involvement of bilateral temporal structures. *Cmap*_*n*_ for the Dorsal Attention network shows large stability increases within itself, involving as well some regions of the posterior DMN and a right anterior frontal region (also lateralized on the side of the focus). A very similar pattern was found for *Cmap*_*n*_ of the posterior DMN network (results not shown). *Cmap*_*n*_ for the Sensory Motor network identified increase in stability with itself, involving as well some frontal more anterior regions, far from the focus (results not shown).

**Figure 8:**
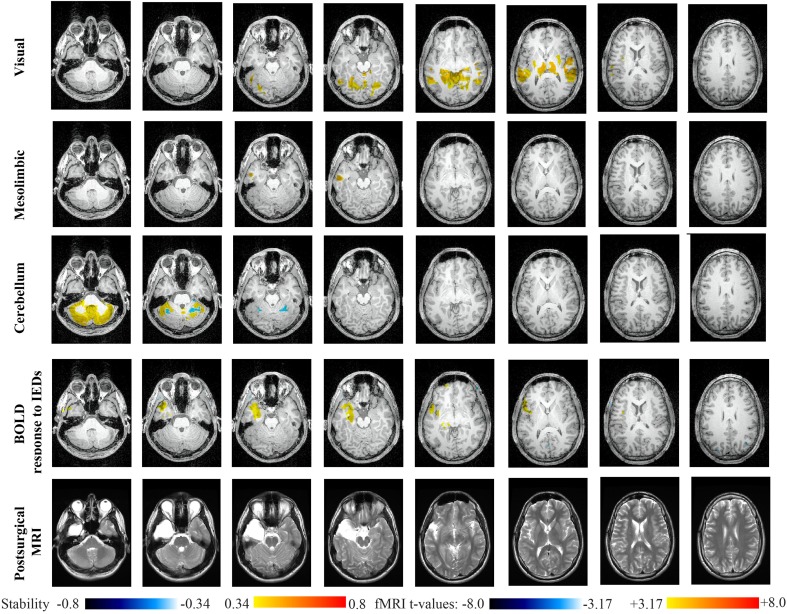
**Evaluation of DANI on Patient 3 with left MTLE.** Results are presenting most salient stability changes observed in *Cmap*_*n*_ followed by a t-map of the BOLD response to epileptic discharges and a postsurgical T2 MRI, all resampled in the native anatomical MRI space of the patient. Most salient stability changes observed for *Cmap*_*n*_ of Visual, Mesolimbic and Cerebellum networks are presented. The Visual and Cerebellum networks were also involved in significant interactions of modularity changes at *p* < 0.001. Same colormap conventions than in Figure 6.

Main results obtained for patient 3 with left MTLE and hippocampal sclerosis are presented in Figure 8. BOLD activations to epileptic discharges were found within the left mesio-temporal focus, involving as well the left temporal neocortex. BOLD results were classified as fully concordant with the location of the resection involving the anterior part of the temporal lobe. Most salient stability changes in *Cmap*_*n*_ were found for the Visual, Mesolimbic, Cerebellum and Dorsal Attention networks. The Visual, Cerebellum, Dorsal Attention and also the Language networks have been identified within significant interaction in modularity changes. Very widespread stability increases have been identified within *Cmap*_*n*_ of the Visual network, involving mainly the secondary-association visual areas including the fusiform gyri bilaterally, and not the primary visual areas. Stability increases were also found in the left hippocampus, bilateral thalami, putamen, insulae, cerebellum and some regions of the Dorsal Attention network. Interestingly, *Cmap*_*n*_ of the Mesolimbic network showed a well-localized and lateralized left temporo-lateral stability increase, closely related to the focus. *Cmap*_*n*_ of the Cerebellum network exhibited some reorganization resulting in stability increases and decreases within the cerebellum itself. *Cmap*_*n*_ of the Dorsal Attention network suggests stability increases within itself, involving also some regions of the posterior DMN (results not shown). *Cmap*_*n*_ of the Language network shows increase in stability in bilateral temporo-posterior regions at the temporo-occipital junction, involving also bilateral fusiform gyri. Increase stability in bilateral thalami was also identified (results not shown).

**Figure 9:**
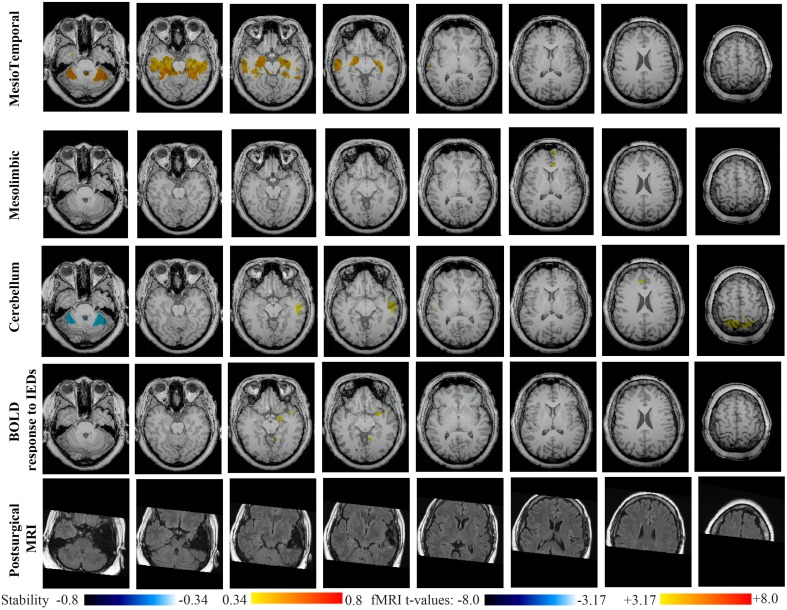
**Evaluation of DANI on Patient 4 with right MTLE.** Results are presenting most salient stability changes observed in *Cmap*_*n*_ followed by a *t*-map of the BOLD response to epileptic discharges and a postsurgical FLAIR MRI, all resampled in the native anatomical MRI space of the patient. Most salient stability changes observed for *Cmap*_*n*_ of Mesio-Temporal and Mesolimbic networks are presented. We also present *Cmap*_*n*_ of the Cerebellum network (max. stability increase of 0.48), since it was involved in significant interactions of modularity changes at *p* < 0.001. Same colormap conventions than in Figure 6.

Main results obtained for patient 4 with right MTLE and hippocampal sclerosis are presented in Figure 9. BOLD activation to epileptic discharge was found in the right mesio-temporal structures, fully concordant with the location of the resection. Most salient stability changes in *Cmap*_*n*_ were found for the Mesio-Temporal, Mesolimbic and Sensory Motor networks, whereas significant interaction in modularity changes were found for the anterior DMN, the Visual, the Cerebellum and the Sensory Motor networks. The largest stability increases in *Cmap*_*n*_ were found for the MesioTemporal network (maximum of 0.7), involving mainly the right mesio-temporal focus, as well as left mesial and lateral temporal regions and bilateral cerebellum. *Cmap*_*n*_ of the Mesolimbic network shows stability increases in the fronto-mesial and polar regions on the side of the focus. *Cmap*_*n*_ for the Cerebellum network shows very interestingly a lateralized stability increase in the right temporal region, very close to the focus, and a bilateral stability decrease within the cerebellum itself. Slight stability increases within regions of the Mesolimbic and Dorsal Attention networks were also observed. Note that even if Cerebellum *Cmap*_*n*_ was not considered among the most salient findings, the maximum stability increase of 0.48 was very close to our arbitrary threshold of 0.5. Within *Cmap*_*n*_ of the Visual network, we identified a bilateral stability increase within secondary-association visual areas including the fusiform gyri and within the right insula (lateralized on the side of the focus, results not shown). *Cmap*_*n*_ of the Sensory Motor network exhibited stability increases withing itself involving also some regions of the posterior DMN and Visual network, bilaterally (results not shown). *Cmap*_*n*_ of the anterior DMN showed a small focal stability increase in the supplementary motor area (results not shown).

**Figure 10:**
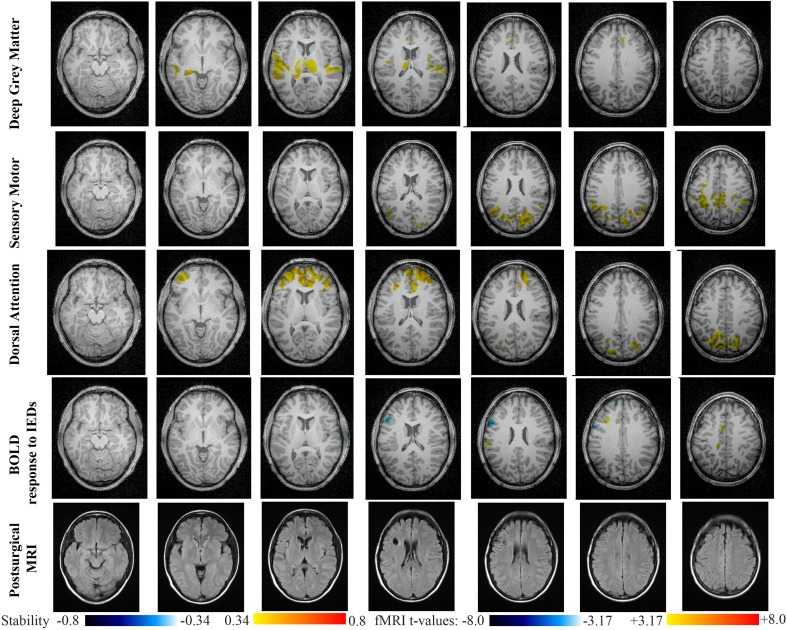
**Evaluation of DANI on Patient 5 with left frontal FCD.** Results are presenting most salient stability changes observed in *Cmap*_*n*_ followed by a *t*-map of the BOLD response to epileptic discharges and a postsurgical FLAIR MRI, all resampled in the native anatomical MRI space of the patient. Most salient stability changes observed for *Cmap*_*n*_ of Deep Gray Matter, Sensory Motor, and Dorsal Attention networks are presented. No networks were involved in significant interactions of modularity changes at *p* < 0.001. Same colormap conventions than in Figure 6.

Main results obtained for patient 5 with left frontal FCD are presented in Figure 10. The resection was circumscribed to the lesional area and a BOLD deactivation response to epileptic discharges had a maximum *t*-value (negative value) at the anterior edge of the resection. Even if the overlapping voxels were only few, the fact that they contained the maximum t-value allowed us classifying this case as “partially concordant.” Most salient stability increases in *Cmap*_*n*_ involved MesioTemporal, Cerebellum, Deep Gray Matter, Sensory Motor and Dorsal Attention networks, whereas no significant interactions of modularity changes could be detected. None of these changes were really spatially concordant with the left frontal focus or lateralized to the side of the focus. *Cmap*_*n*_ of the Deep Gray Matter network exhibited increase stability within itself also involving bilateral insulae. *Cmap*_*n*_ of the Sensory-Motor network shows increase stability within itself and also involving regions of the posterior DMN. *Cmap*_*n*_ of the Dorsal Attention network shows increase stability within itself and in some bilateral lateral and mesial frontal regions of the anterior DMN. *Cmap*_*n*_ of the MesioTemporal network shows increase in stability in bilateral Cerebellum regions and *Cmap*_*n*_ of the Cerebellum shows increase in stability in bilateral temporal (results not shown).

**Figure 11:**
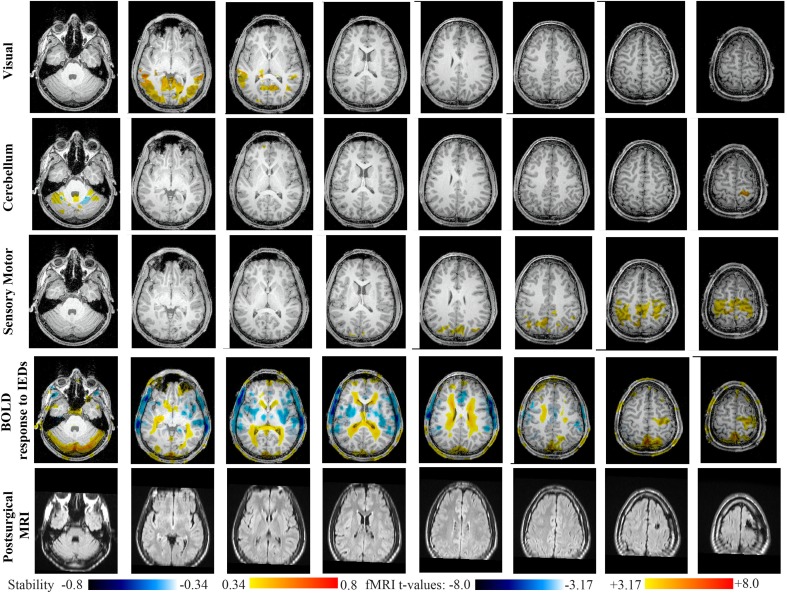
**Evaluation of DANI on Patient 6 with right frontal FCD.** Results are presenting most salient stability changes observed in *Cmap*_*n*_ followed by a *t*-map of the BOLD response to epileptic discharges and a postsurgical FLAIR MRI, all resampled in the native anatomical MRI space of the patient. Most salient stability changes observed for *Cmap*_*n*_ of Visual, Cerebellum, and Sensory Motor networks are presented. The Visual and Cerebellum networks were also involved in significant interactions of modularity changes at *p* < 0.001. Same colormap conventions than in Figure 6.

Main results obtained for patient 6 with right frontal FCD are presented in Figure 11. The resection was circumscribed to the lesional area. The BOLD response to epileptic discharges was really noisy. The cluster of BOLD activation showing a maximum *t*-value in the right central region is partially concordant with the edge of resection, but the presence of motion artifacts cannot let us classify this case as “concordant.” Most salient stability changes in *Cmap*_*n*_ involved MesioTemporal, Auditory, Visual, Mesolimbic, Cerebellum, and Sensory Motor networks. Among these networks, the Visual, Mesolimbic and Cerebellum networks were also involved in significant interactions of modularity changes. *Cmap*_*n*_ of the visual network shows stability increase within secondary-association visual areas including the fusiform gyri. *Cmap*_*n*_ of the Cerebellum network exhibited local stability increases and decreases within the Cerebellum but also a very focal and very intense right postcentral stability increase (maximum increase of 0.72), partially concordant with BOLD activation and lateralized to the side of the lesion. *Cmap*_*n*_ of the Sensory Motor network shows increase stability within itself and involving some more posterior parietal bilateral regions. *Cmap*_*n*_ of the Mesolimbic network shows increase stability within bilateral temporal regions, *Cmap*_*n*_ of the Mesio-Temporal network shows increase stability in bilateral temporal and anterior cingulate region (part of the Mesolimbic network) and *Cmap*_*n*_ of the auditory network shows bilateral increase in stability within itself and within Thalami (results not shown). Since BOLD results to epileptic discharges were really contaminated by motion artifacts, similar artifact could have also biased DANI results in this case, although we carefully removed all the frames showing a displacement of more than 0.5 mm as suggested by Power et al. (2012).

Even though we reported mainly the concordance of the stability changes detected using DANI and the BOLD responses to epileptic discharges with the resected area, it is important to point out that for most patients both results, i.e., DANI and BOLD responses to epileptic discharges, were not only showing significant changes closely related to the presumed epileptic focus, but were also exhibiting more complex connectivity patterns reorganization extending to some more distant regions.

## Discussion

The advantage of our proposed method DANI is the ability to identify atypical and individual modular organization of FC for one specific individual. Besides extending BASC (Bellec et al., 2010) to produce trimmed stability maps focussing on the core of the networks, we reproduced the main CRSNs in agreement with previous studies, using the group level BASC analysis on 25 healthy controls (Raichle et al., 2001; Damoiseaux et al., 2006; Fox and Raichle, 2007; Smith et al., 2009). We notably reproduced group level BASC results presented in Bellec et al. (2010), using another database of 25 healthy controls. The trimmed stability maps, representing at the group or at the individual the statistically most stable networks allows focussing on RSN cores rather than on full networks. Our evaluation using simulated data demonstrated than an optimal sensitivity contrast was obtained when choosing a core size μ of 50%. Using these maps, we were able to characterize individual variability with a good compromise between flexibility and consistency. This approach provides consistency across subjects while allowing for flexibility to adapt the networks at the individual level. The combined maps *Cmap*_*n*_ exhibiting significant changes in stability, allowed us to avoid the problem of large and unstable *Z*-scores in regions where the mean and standard deviation of stability in the controls were close to zero. In order to detect abnormal networks statistically stable at the level of individuals, we proposed DANI, which involves the following steps: (i) generation of trimmed stability maps of each network at the individual level, (ii) assessment of significant stability variations in FC using *Cmap*_*n*_, (iii) non-parametric test to automatically detect significant interactions of modularity changes with other CRSNs.

DANI was first evaluated using realistic simulated data. Our results demonstrate that detecting modular changes over various spatial extents was possible. DANI is sensitive to changes caused by the addition of structured signal to modify modular structures. Whatever was the size of the simulated perturbation, DANI detected changes in modularity up to an energy ratio of 7 dB between the original signal and the simulated signal. The criterion to choose an optimal core size μ for the trimmed map is to be sensitive to small changes without completely changing the modular structure, when the affected regions were representing a substantial fraction of the original module. A core size μ of 50% of the most stable regions in each cluster has proven to be a good candidate to meet this criterion. We showed that when the perturbed area was spatially limited (1/6th of the networks), the modular reorganization tended to associate that perturbed zone with one of the two original networks (the auditory network), while removing it from the other one (the somato-sensory network). When increasing the size of that perturbation zone, the new perturbed zone started to overcome the stability of the two original networks, resulting in the extinction of one or both of the original networks.

We acknowledge the fact that the proposed method includes a series of parameters that have to be set, for which the default (recommended) values are summarized in Table 3. First of all, BASC and DANI have been applied on a parcellation of the brain in *R* = 739 ROIs. These ROIs were obtained using a region-growing algorithm proposed in Bellec et al. (2006), in order to ensure homogeneity, over all control subjects, of the measurements on small functional units or parcels. Although a target subject to be evaluated using DANI might exhibit slightly different functional parcels in theory, we believe that those changes will be reflected in the stability strengths of the regions in question. We therefore think that this approach should allow sufficient flexibility to capture individual changes while maintaining a good consistency across subjects. Moreover, the main reason for applying BASC and DANI on a parcellation of the brain was for dimensionality reduction purposes, in order to limit the computational burden of the proposed method. A potential improvement not explored in this study would be to apply BASC and DANI directly at the voxel-level instead of the parcel-level. This interesting modification of the method was out of the scope of the present study. Secondly, the overall analysis was proposed using a scale of *N* = 12 CRSNs, in order to be in agreement with the literature describing those CRSNs (Damoiseaux et al., 2006; Smith et al., 2009). However, in BASC, Bellec et al. (2010) suggested and evaluated an optimization strategy to estimate the parameters *k*, *L* and *N* for different scales of interest. In this context, it would be highly relevant in a future study to investigate how the choice of such a scale could impact the sensitivity and specificity of DANI results. Finally, when presenting these preliminary results on 6 patients with focal epilepsy, we focussed our interest on the most salient findings only (cf. *Zmask*_*n*_ threshold at 3.17 (*p* < 0.001 non-corrected), *Cmap*_*n*_ threshold at 0.34 (99.9% percentile over the control population and maximum *Cmap*_*n*_ showing more than 50% of stability changes). It would be relevant to investigate furthermore in a future study, the specificity of the method when applied on a larger dataset of controls and patients as well as the reproducibility of the results, using test/re-test reliability for instance.

**Table 3:** **Parameters used in the method**.

**Symbol**	**Description**	**Value(s)**	**Default**
-	Maximal size of a region in the region-growing process	800 mm^3^	800–1000 mm^3^
R	Number of regions	739	This was obtained by the region-growing on all controls
k	Individual level clustering threshold	13	Estimated using BASC
L	Group level hierarchical clustering threshold	14	Estimated using BASC
N	Final clustering threshold at the group level	12	Selected scale in agreement with most literature on CRSNs
μ	Core size for estimating the trimmed stability maps	25%, 50%, 75%	50% according to our simulations
-	*Zmask*_*n*_ threshold to identify the mask of significant stability changes (*p* < 0.001, non-corrected)	3.17	3.17
-	*Cmap*_*n*_ threshold (combined map assessing the amount of stability change), selected as the 99.9% percentile estimated over the control population	0.34	
-	Threshold for the detection of most salient findings: max |*Cmap*_*n*_| > threshold	0.5	0.5
-	Significant level testing for interaction of modularity changes between network *n* and CRSN *j*: λ_*n,j*_ (non-parametric test)	*p* < 0.001	*p* < 0.001

List of the parameters that are referred in the method with their respective values as well as the default recommended values.

DANI was then evaluated on resting state fMRI data from six patients with focal epilepsy who underwent simultaneous EEG/fMRI acquisition followed by surgery. Only patients with a seizure free outcome and at least 12 months follow up were selected. For all patients, the BOLD responses to epileptic discharges were evaluated as fully or partially concordant with the location of the resection, following the methodology proposed by An et al. (2013). Interpretation of BOLD responses for patient 5 and 6 was more difficult (details hereunder). The most significant BOLD responses (maximum positive or negative *t*-values) were considered to assess the level of concordance between BOLD results and the location of the resection. Whereas we acknowledge that BOLD responses are also often found distant from the presumed focus (e.g., patients 1, 2, and 6), we previously demonstrated that considering the most significant BOLD results provided best agreement with the presumed focus and EEG results (Pittau et al., 2012a; Heers et al., 2014). Overall DANI identified clearly several outlier networks for each patient. These changes in the stability of FC patterns were salient, showing increases in stability larger than 0.5, whereas the 99.9% percentile of stability increase measured over the healthy controls database was 0.34. For 5 out of 6 patient, “abnormal” or outlier networks closely related to the epileptogenic focus were detected. We also found reorganizations of some remote networks distant from the focus (e.g., Dorsal Attention network and posterior DMN). These results suggest large reorganization of FC patterns, extended far the from the focus. Similar findings have been suggested in group analysis of MTLE patients (see Bernhardt et al., 2013; for a recent review), but also in the few studies including individual level analysis of patients with epilepsy (Negishi et al., 2011; Stufflebeam et al., 2011; Luo et al., 2014). Whereas CRSNs observed in healthy controls are usually bilateral, we identified for 5 out of 6 patients at least one abnormal network exhibiting increase in stability lateralized on the side of the focus. Despite widespread involvement of several networks, the importance of laterality in FC patterns of patients with epilepsy has been suggested as a key feature by several studies (Bettus et al., 2009; Negishi et al., 2011; Constable et al., 2013; Luo et al., 2014). For instance, Negishi et al. (2011) proposed to use patient specific BOLD responses to epileptic discharges to define seeds for a seed-based FC analysis. They found that poor surgical outcome was associated with a low degree of laterality of FC maps. The potential clinical impact of providing accurate and sensitive FC analysis during presurgical investigation in the context of neurooncology, epilepsy surgery, and deep brain stimulation has been recently reviewed by Lang et al. (2014), pointing out the importance of developing methods dedicated to single subject analysis of FC patterns.

For patients 1 and 2 who had right orbito-frontal epilepsy, DANI detected specific reorganization within the Mesolimbic, Mesio-Temporal and Cerebellum networks. The right orbito-frontal focus belongs to the Mesolimbic network, which exhibited stability increases in the Mesolimbic and Mesio-Temporal networks. Interaction between these two networks was not surprising. Concerning the involvement of the cerebellum, BOLD responses in cerebellum regions during frontal epileptic discharges have been suggested by Fahoum et al. (2012). Several studies have demonstrated the interaction among brain regions belonging to the Mesolimbic and Mesio-Temporal networks. This topic has been recently reviewed for TLE (Cataldi et al., 2013), but it is much more difficult to establish connectivity starting from the orbito-frontal region. Whereas the epileptogenic network in TLE is relatively well-characterized (Spencer, 2002) and encompasses orbito-frontal regions, it is not clear which brain regions should be part of the orbito-frontal network in epilepsy. Nevertheless, intracranial EEG studies in orbito-frontal epilepsy showed that epileptic discharges from the orbito-frontal focus have the tendency to spread toward the mesial temporal structures (Munari et al., 1995; Smith et al., 2004).

For patients 3 and 4 with, respectively left and right MTLE and hippocampal sclerosis, the most salient findings identified using DANI involved mainly the Visual, the Mesolimbic and the Cerebellum networks, as well as the Mesio-Temporal network (patient 4 only). As stated before, the connection and propagation pathways between the Mesio-Temporal and the Mesolimbic regions have been clearly identified in MTLE patients (Spencer, 2002) and associated changes in FC involving these networks in MTLE patients have been identified by our group Pittau et al. (2012b) and carefully reviewed in Cataldi et al. (2013) and Bernhardt et al. (2013). The involvement of BOLD activation in the mid-cingulate gyri, at the time of temporal lobe discharges, has been demonstrated by Fahoum et al. (2012). Importantly, DANI identified clearly lateralizing results, as for instance a left temporal increase in stability observed for patient 3 within the Mesolimbic *Cmap*_*n*_, and a right temporal increase in stability observed for patient 4 within the Cerebellum *Cmap*_*n*_. The importance of laterality patterns in FC studies in MTLE has been suggested in Bettus et al. (2009) showing increased connectivity between temporal regions contralateral to the focus and in Pittau et al. (2012b) showing decreased connectivity between ipsilateral and contralateral temporo-mesial regions. Interestingly, the Visual network *Cmap*_*n*_ was detected by DANI for both patients. Stability increases consisted mainly in lateral parts of this network, containing the fusiform gyri. These regions are secondary-association visual and memory areas and are connected to the posterior part of mesial and lateral part of the temporal lobes. Note that for patient 3, stability increases in Visual network *Cmap*_*n*_ were also found in subcortical sutructures (thalamus and putamen).

Overall results obtained for patients 5 and 6 were less obvious to interpret. Differently from the first four cases, some concerns were raised regarding the BOLD responses to epileptic discharges: case 5 had a deactivation only partially concordant with the location of the resection; in case 6 the whole BOLD response was affected by motion artifacts, and only a part of the activation was found in “partial” agreement with the resected area. In both cases, the lesion consisted in a relatively focal dysplasia clearly identified on structural MRI data and the resection was circumscribed to the lesion. Some interesting findings were observed for patient 6, showing some partial concordance between a right postcentral increase in stability observed for the Cerebellum network *Cmap*_*n*_, stability increase within the sensory motor network, a right central BOLD activation and a right precentral lesion. However, the fact that a small resection allowed these patients to become seizure free suggests that the network reorganization was less spread spatially, despite clear involvement of the sensory-motor network for patient 6.

For 4 out of 6 patients, DANI detected the Dorsal Attention network as abnormal, showing mainly stability increases within itself but also in posterior and anterior DMN regions and Mesolimbic regions. Overall, these network reorganizations were bilateral and distant from the focus. Whereas an involvement of DMN is well-known for patients with temporal and extratemporal lobe epilepsy (Laufs et al., 2007; Kobayashi et al., 2009; Fahoum et al., 2012), the attention network has been less studied, especially in patients with extratemporal lobe epilepsy, probably because of the difficulty of finding homogeneous groups for this type of epilepsy. Nevertheless, the involvement of the dorsal attention network has been demonstrated as impaired in patients with TLE (Zhang et al., 2009), frontal epilepsy (Fahoum et al., 2012) as well as in patients with epileptic syndromes (Vaudano et al., 2014).

In the last decade connectivity studies have shed light to several aspects of the epileptic brain. However, clinical applications (for diagnostic or prognostic purposes) of each method, including our proposed method DANI, require further validations before being consistently applied to the clinical management of the single patient. Moreover, it is important to remember that each diagnostic technique has to be integrated with all the other clinical and diagnostic data of the individual patient.

## Conclusion

We proposed DANI as a new method to capture inter-individual variations in RSNs, and assess its performance in realistic simulations and its potential usefulness in patients. DANI is based on an extension of the BASC method to extract FC networks, allowing the assessment of statistical stability in RSNs at the individual level. Our results suggest that the ability of the method to capture modular changes is affected by the core size used to obtain the trimmed map. BASC is indeed sensitive to modular changes within the FC structure of a subject and DANI is able to detect small perturbations of those modules as well as the fusion of areas of various sizes with good sensitivity. The evaluation of the method on subjects with epilepsy identified in most cases (5/6) abnormal networks exhibiting significant changes in FC stability closely related and lateralized to the epileptogenic focus. These results are encouraging since the findings are supported by other modalities and were obtained without any prior on the disease. DANI also showed the involvement of distant networks, not containing the focus, suggesting remote reorganization. Although the fact that focal epilepsies affect distant networks is more and more recognized (Richardson, 2012), it is still premature to evaluate whether significant changes in FC are linked to effects of the discharge of the individual patient, or to other effects more remotely associated to the epilepsy of the patient (e.g., effect of medication, neuropsychological impairment). Clinical studies involving more patients and a specific comparison with the epileptogenic network of each patient will be required to investigate these issues.

### Conflict of interest statement

The authors declare that the research was conducted in the absence of any commercial or financial relationships that could be construed as a potential conflict of interest.

## Footnotes

^1^NIAK website: http://www.nitrc.org/projects/niak.

## Supplementary material

Figure S1 **Data workflow used to generate the combined significant stability-map *Cmap*_*n*_ comparing, for one particular network, the trimmed stability map of one subject vs. the population of controls**. A binary mask *Zmask*_*n*_ assessing significant changes in local stability is first estimated. This binary mask is then applied to the subject trimmed stability map centered using the mean stability of all controls. *Cmap*_*n*_ allows the identification of most stable regions showing significant changes in stability when compared to the average of controls. In this example, *Cmap*_*n*_ of the auditory network identified an increase in stability in bilateral Thalami. Note that the most posterior region also identified in *Zmask*_*n*_ was not detected in *Cmap*_*n*_, because it was associated with very low stability values.

Figure S2 **Method used to combine some structured noise time-series with the original fMRI signal of the sensory-motor and auditory network time-series**. This process is repeated for each region of these two networks located inside the red area corresponding to the simulated perturbed zone. The structured noise consisted in the averaged time-series of the visual network of an independent control, thus introducing additional correlations between the auditory and sensory-motor networks.
