# Supplementary figures and images for: Detection of abnormal resting-state networks in individual patients suffering from focal epilepsy: an initial step toward individual connectivity assessment
Source: Front Neurosci. 2014 Dec 23;8:419. doi: 10.3389/fnins.2014.00419 (PMC4274904; doi:10.3389/fnins.2014.00419)

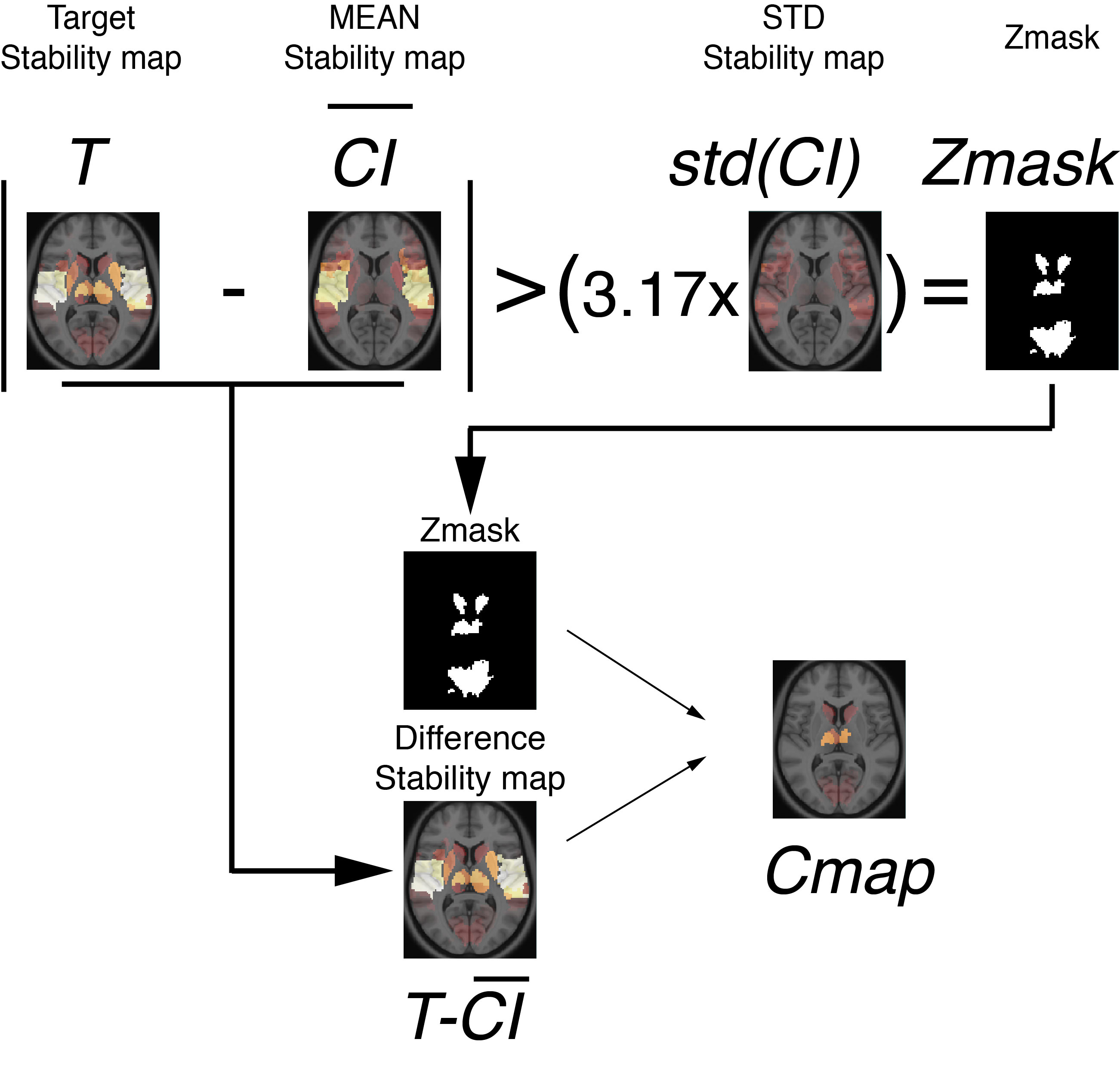

Supplement: Figure S1 — Data workflow used to generate the combined significant stability-map Cmapn comparing, for one particular network, the trimmed stability map of one subject vs. the population of controls. A binary mask Zmaskn assessing significant changes in local stability is first estimated. This binary mask is then applied to the subject trimmed stability map centered using the mean stability of all controls. Cmapn allows the identification of most stable regions showing significant changes in stability when compared to the average of controls. In this example, Cmapn of the auditory network identified an increase in stability in bilateral Thalami. Note that the most posterior region also identified in Zmaskn was not detected in Cmapn, because it was associated with very low stability values. [file Image1.JPEG]

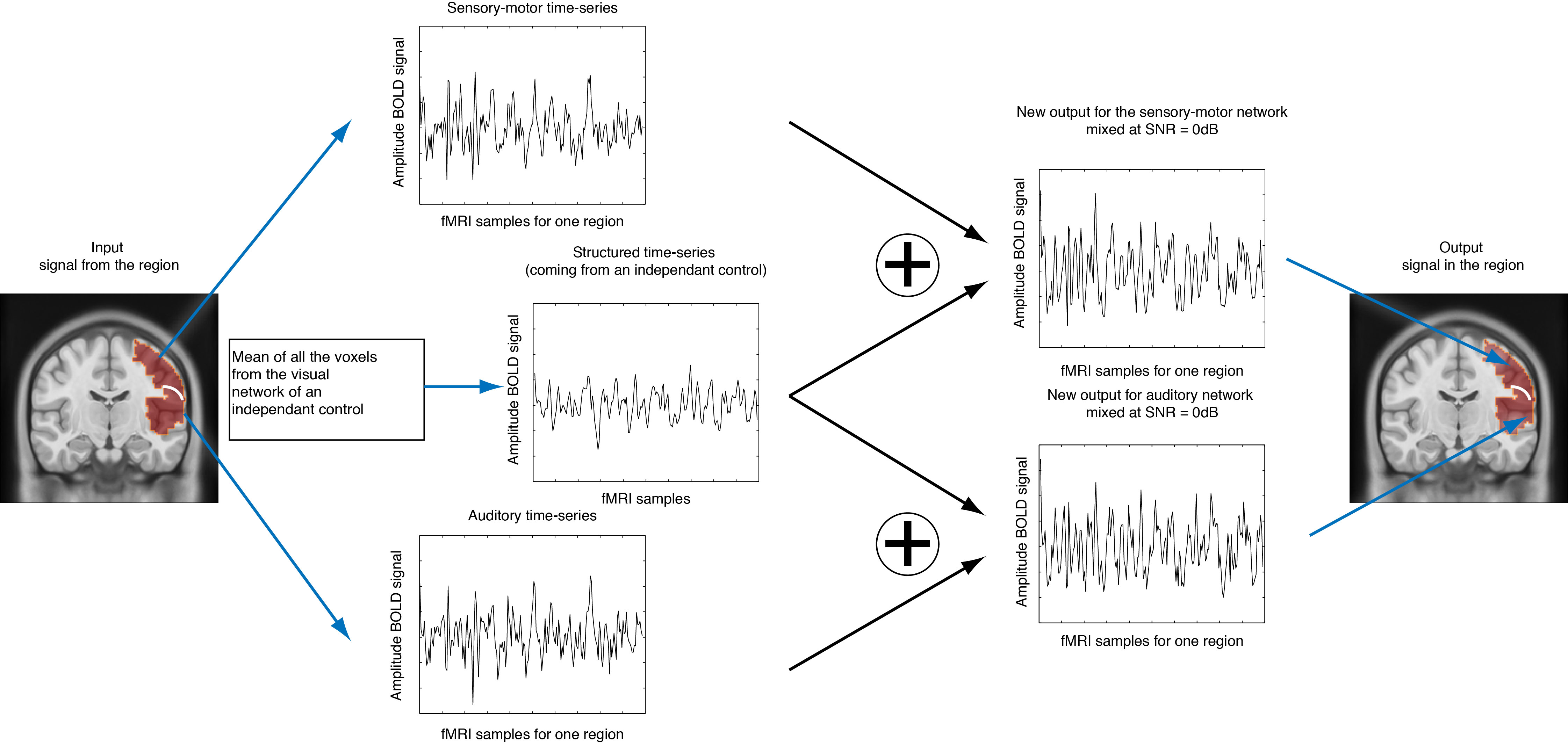

Supplement: Figure S2 — Method used to combine some structured noise time-series with the original fMRI signal of the sensory-motor and auditory network time-series. This process is repeated for each region of these two networks located inside the red area corresponding to the simulated perturbed zone. The structured noise consisted in the averaged time-series of the visual network of an independent control, thus introducing additional correlations between the auditory and sensory-motor networks. [file Image2.JPEG]
